# Supplementary material for: Single-cell transcriptomics reveals the effect of PD-L1/TGF-β blockade on the tumor microenvironment
Source: BMC Biol. 2021 May 25;19:107. doi: 10.1186/s12915-021-01034-z (PMC8147417; doi:10.1186/s12915-021-01034-z)
Supplement: Supplementary file 1 — Additional file 1. Supplementary figures. Figure S1. Anti-PD-L1 plus anti-TGF-β in the murine tumor model HuPD-L1-MC38. Figure S2. Anti-PD-L1 plus anti-TGF-β in the murine tumor model CT26. Figure S3. Quality control of single cell RNA-seq of anti-PD-L1 ±anti-TGF-β treated EMT6 tumor-bearing mice. Figure S4. CD45-cell analysis and InferCNV of anti-PD-L1 ±anti-TGF-β treated EMT6 tumor-bearing mice. Figure S5. scRNA-seq marker gene expression. Figure S6. scRNA-seq cell type composition and chemokine expression. Figure S7. Association of chemokine gene expression with inferred immune cell infiltration in TCGA. Figure S8. Flow cytometry-based expression of key genes in tSNE identified populations. [file 12915_2021_1034_MOESM1_ESM.pdf]

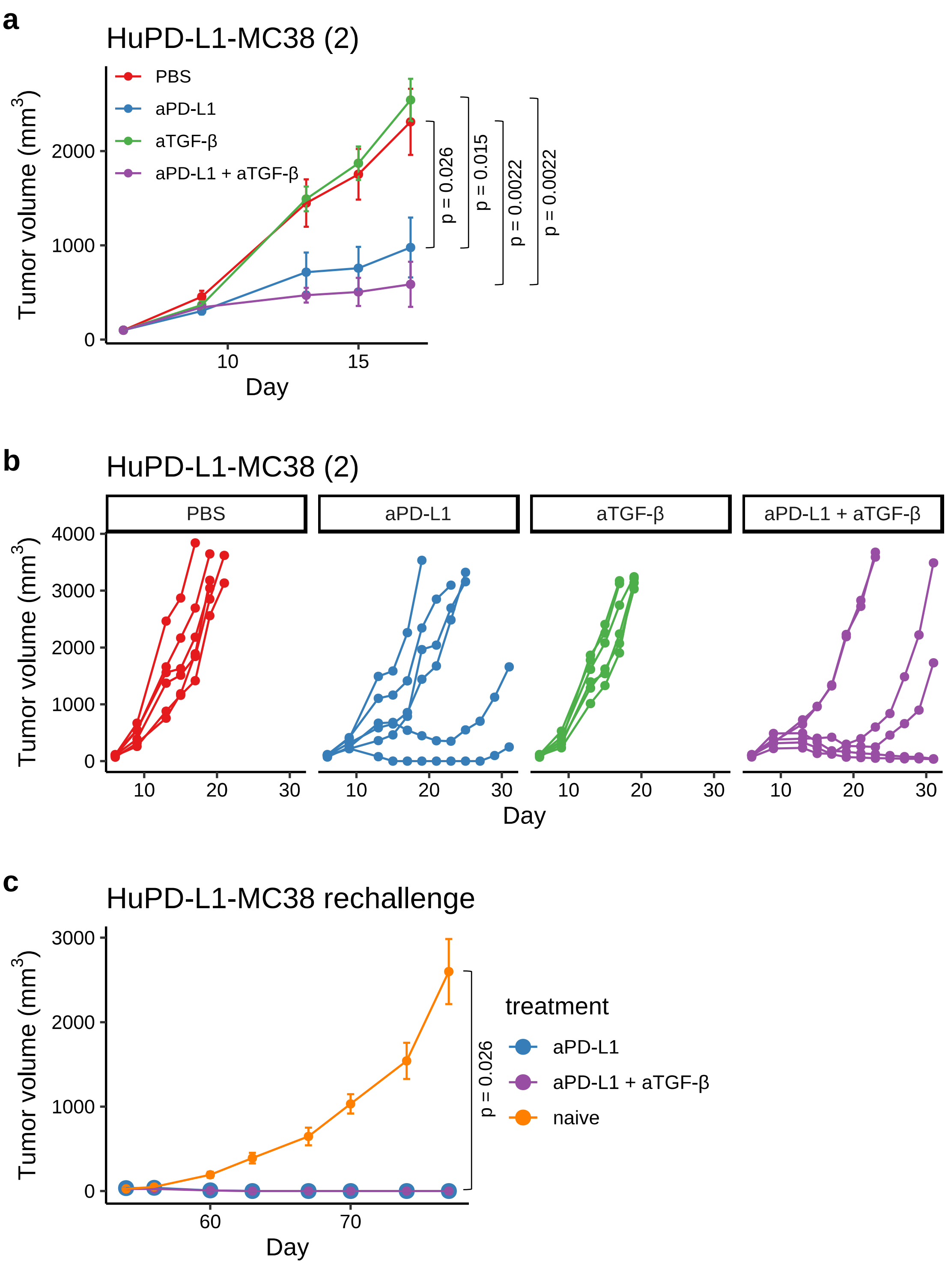

Figure S1. Anti-PD-L1 plus anti-TGF-β in the murine tumor model HuPD-L1-MC38. (a-b) Mice bearing s.c. HuPD-L1-MC38 tumors (n = 6 per group) were dosed I.P. biweekly for three weeks with PBS, aPD-L1 (2 mg/kg; atezolizumab), aTGF-β (10 mg/kg; 1D11), or aPD-L1 plus aTGF-β. **(a)** Average MC38 tumor volume ± SEM is shown. P-values were determined using Wilcoxon rank sum test, comparing tumor sizes on day 17. Non-significant p-values are not shown. **(b)** Spider plots showing tumor volume for individual mice over time. **(c)** Mice from Figure 1a that resulted in a complete response (aPD-L1, n = 1; aPD-L1 + aTGF-β, n = 3) were re-challenged with s.c. HuPD-L1-MC38 tumor cells at the opposite flank. Treatment naïve, wildtype mice (n = 6) were used as a control. Average tumor volume ± SEM is shown. P-value was determined using Wilcoxon rank sum test, comparing tumor sizes on day 77 between naïve and aPD-L1 + aTGF-β groups.

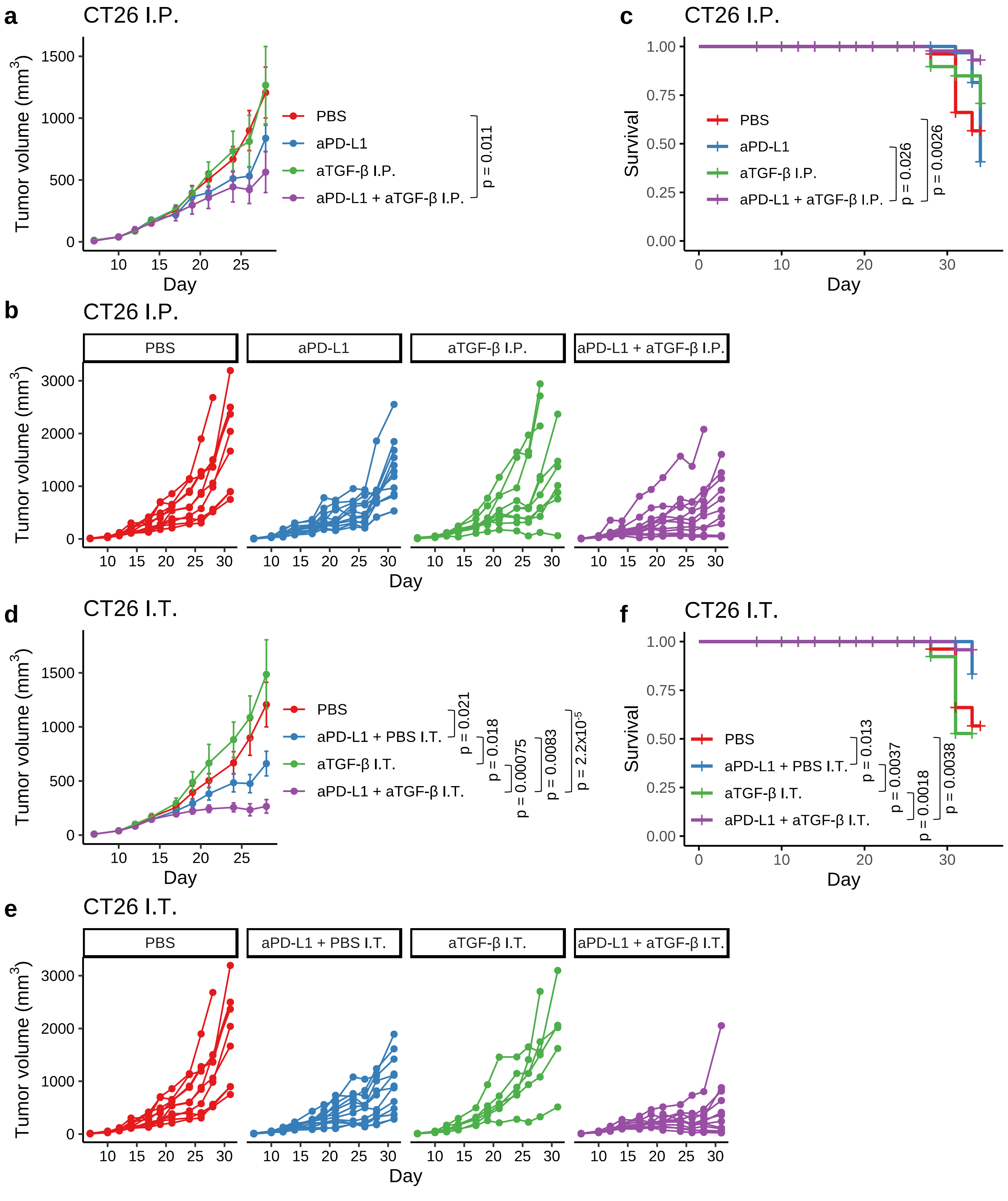

Figure S2. Anti-PD-L1 plus anti-TGF-β in the murine tumor model CT26. (a-c) Mice bearing CT26 tumors were dosed intraperitoneally (I.P.) two times a week for 3 weeks with PBS (n = 10), aPD-L1 (2 mg/kg; atezolizumab; n = 12), aTGF-β (10 mg/kg; 1D11; n = 10), or aPD-L1 + aTGF-β (n = 12). **(a)** Average tumor volume ± SEM is shown. P-value was determined using Wilcoxon rank sum test, using tumor sizes on day 28. **(b)** Spider plots showing CT26 tumor volume for individual mice over time. **(c)** Survival plot for the study. P-values were determined using log-rank test. **(d-f)** Mice bearing CT26 tumors were dosed two times a week for 3 weeks with PBS (I.P.; n = 10; same PBS arm as in **a-c**), aPD-L1 (2 mg/kg; atezolizumab; I.P.) + PBS (intratumorally, I.T.) (n = 12), aTGF-β (10 mg/kg; 1D11; I.T.; n = 6), or aPD-L1 (I.P.) + aTGF-β (I.T.) (n = 12). **(d)** Average tumor volume ± SEM is shown. P-values were determined using Wilcoxon rank sum test, using tumor sizes on day 28. **(e)** Spider plots showing CT26 tumor volume for individual mice over time. **(f)** Survival plot for the study. P-values were determined using log-rank test. Non-significant p-values are not shown for all panels.

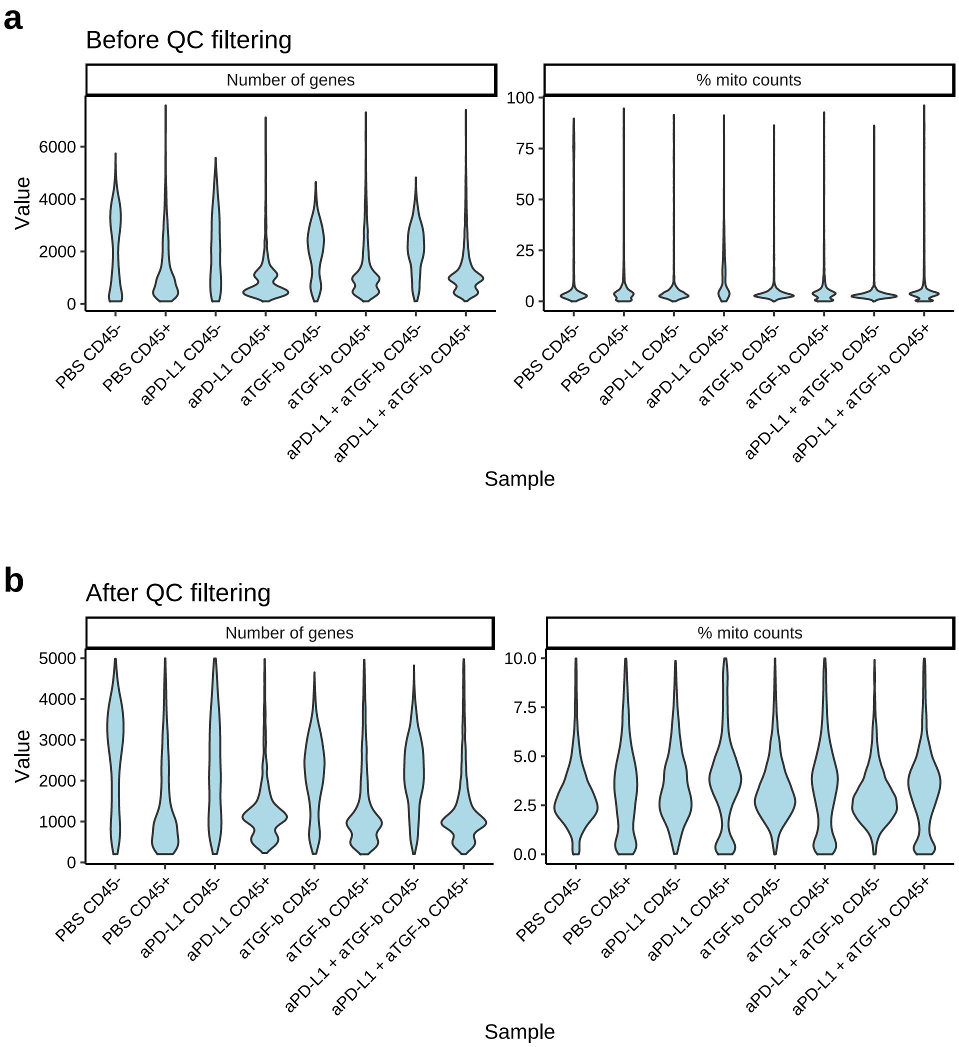

Figure S3. Quality control of single cell RNA-seq of anti-PD-L1 ± anti-TGF-β treated EMT6 tumor-bearing mice. (a) Violin plots showing the distribution of the number of genes (left) and percent read count that mapped to mitochondrial genes (right) per cell, before removing low quality cells. **(b)** The same plots after removing cells with more than 10% mitochondrial reads and fewer than 200 or more than 5,000 expressed genes, as low and high number of gene counts may indicate low quality cells and cell multiplets, respectively.

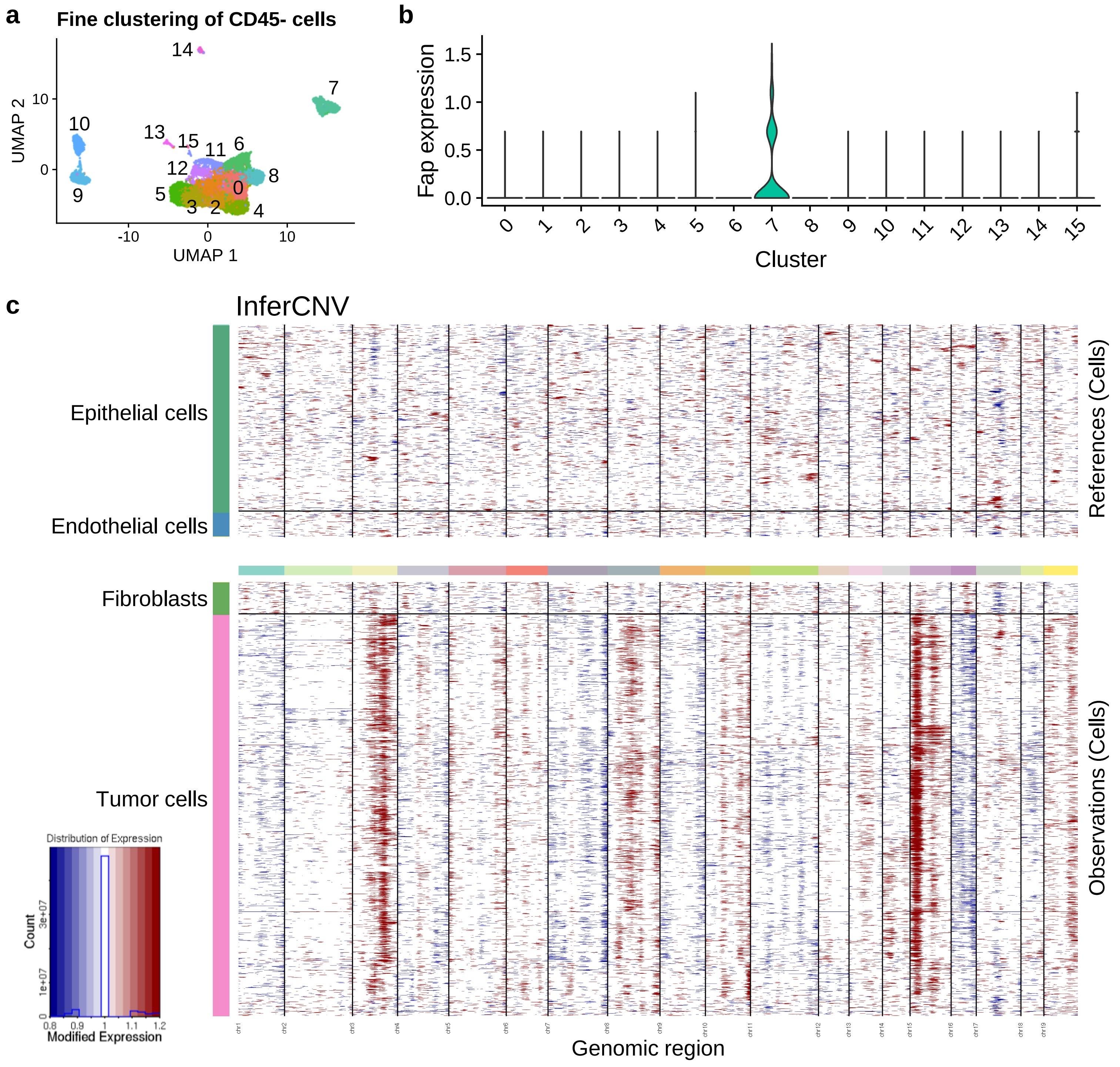

Figure S4. CD45- cell analysis and InferCNV of anti-PD-L1 ± anti-TGF-β treated EMT6 tumor-bearing mice. (a) Single cell transcriptomes for all CD45- cells visualized on a UMAP plot. Cell clusters are colored and numbered. **(b)** Violin plot showing *Fap* expression level in the different CD45- cell clusters. **(c)** Inferred copy number for individual cells of the different CD45- cell types (y-axis) along the mouse genome (x-axis). Regions with gain and loss of copy number are shown in red and blue, respectively**.**

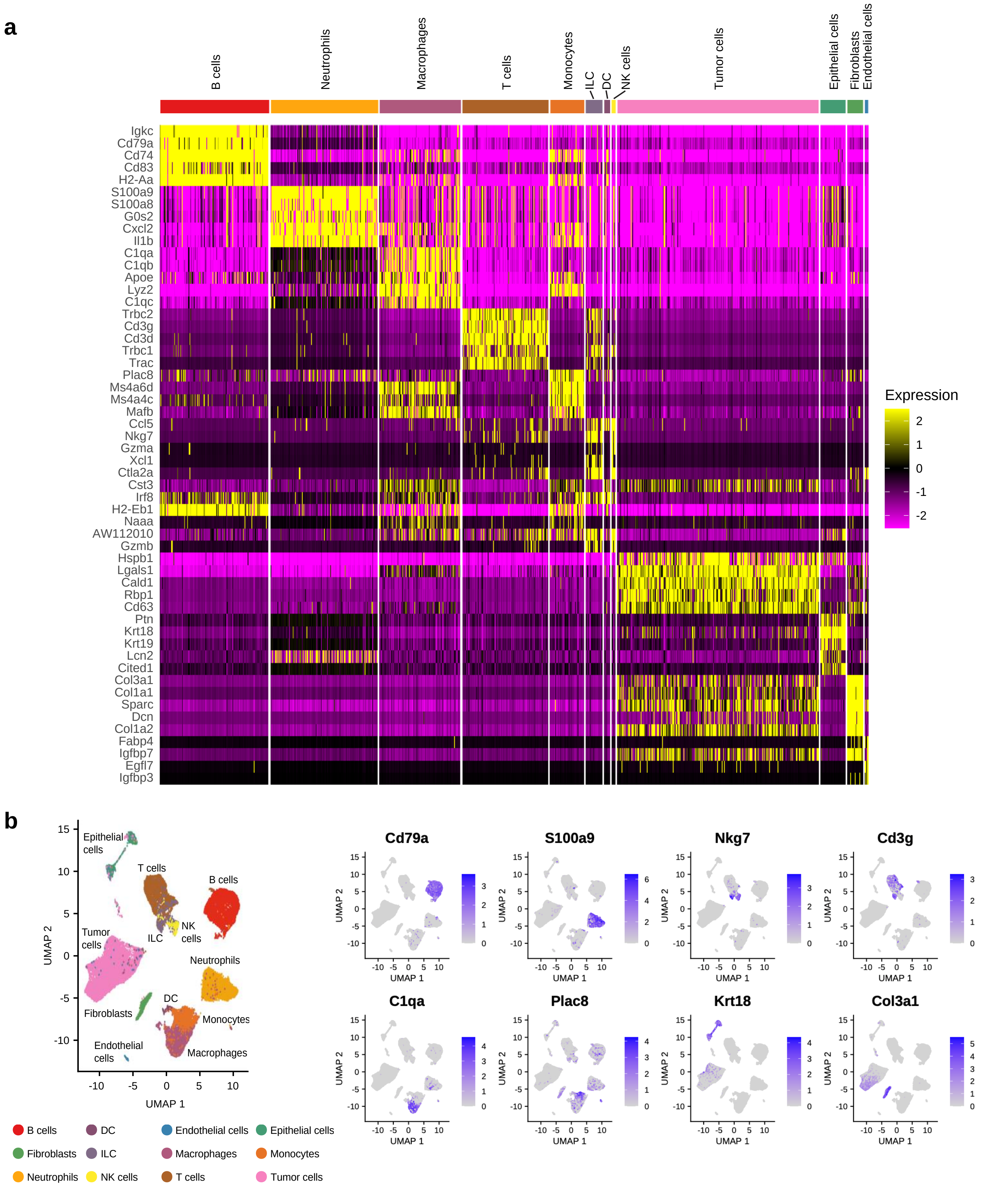

Figure S5. scRNA-seq marker gene expression. (a) Heatmap showing relative gene expression of top marker genes most differentially expressed in each cell type relative to other cell types. **(b)** Left: Single cell transcriptomes for all cells visualized on a UMAP plot (same data as from Figure 2a). Right: Relative expression of selected marker genes displayed on the UMAP plot.

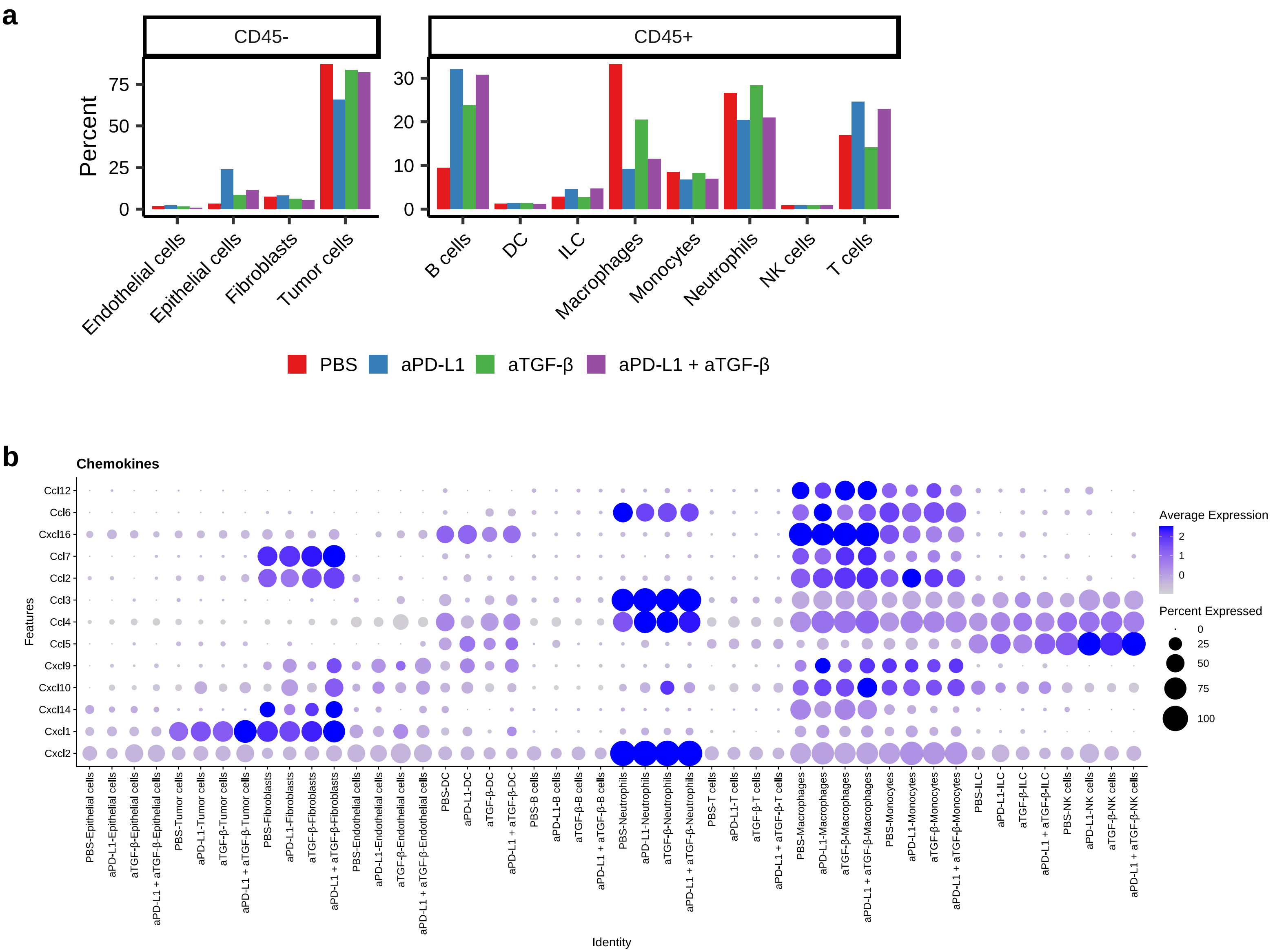

Figure S6. scRNA-seq cell type composition and chemokine expression. (a) Percent composition of cells types within the CD45- cells (left) and the CD45+ (right) cells. The different treatment groups are shown in different colors, as indicated in the legend. **(b)** Dot plot showing chemokine gene expression in different cell types under different treatments. The size of the dots indicates percent cells expressing the chemokine gene, while the color of the dots indicates average gene expression level.

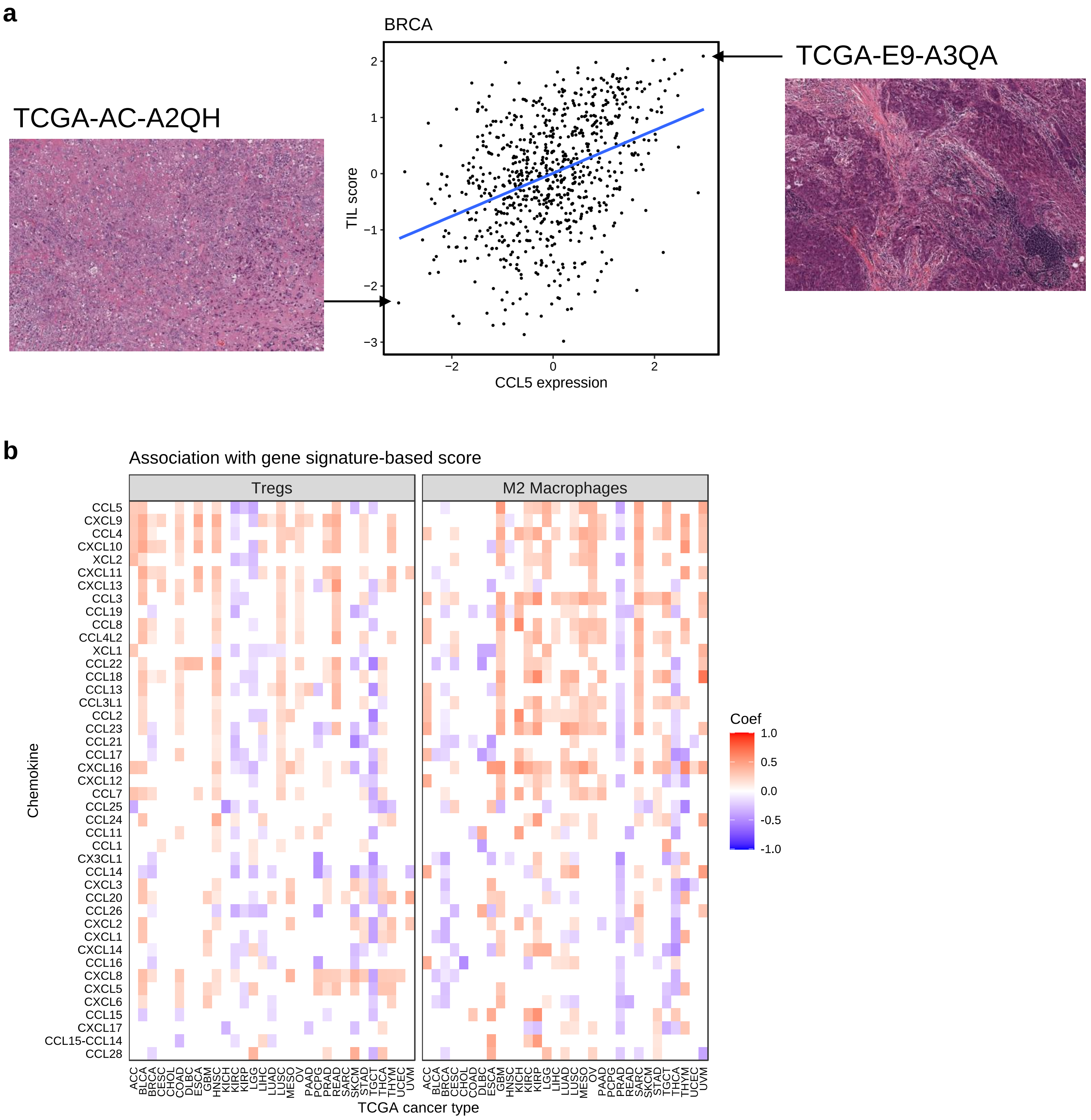

Figure S7. Association of chemokine gene expression with inferred immune cell infiltration in TCGA. (a) The scatter plot shows the correlation between *CCL5* expression and pathology image-based tumor-infiltrating lymphocyte (TIL) score in breast cancer (BRCA) in TCGA. Each point represents a tumor sample. Both axes are shown in Z-score space. TIL score is inferred computationally from H&E-stained pathology images of the tumor samples. Representative images for a high TIL sample and a low TIL sample are shown. **(b)** Heatmap showing association of chemokines with gene signature-based immune scores for regulatory T cells (Tregs) and M2 macrophages. The order of the chemokines on the y-axis are as shown in Figure 4c, while the x-axis indicates TCGA cancer types. The color indicates regression coefficients from the linear models (red/orange = positive association between chemokine expression and immune infiltration).

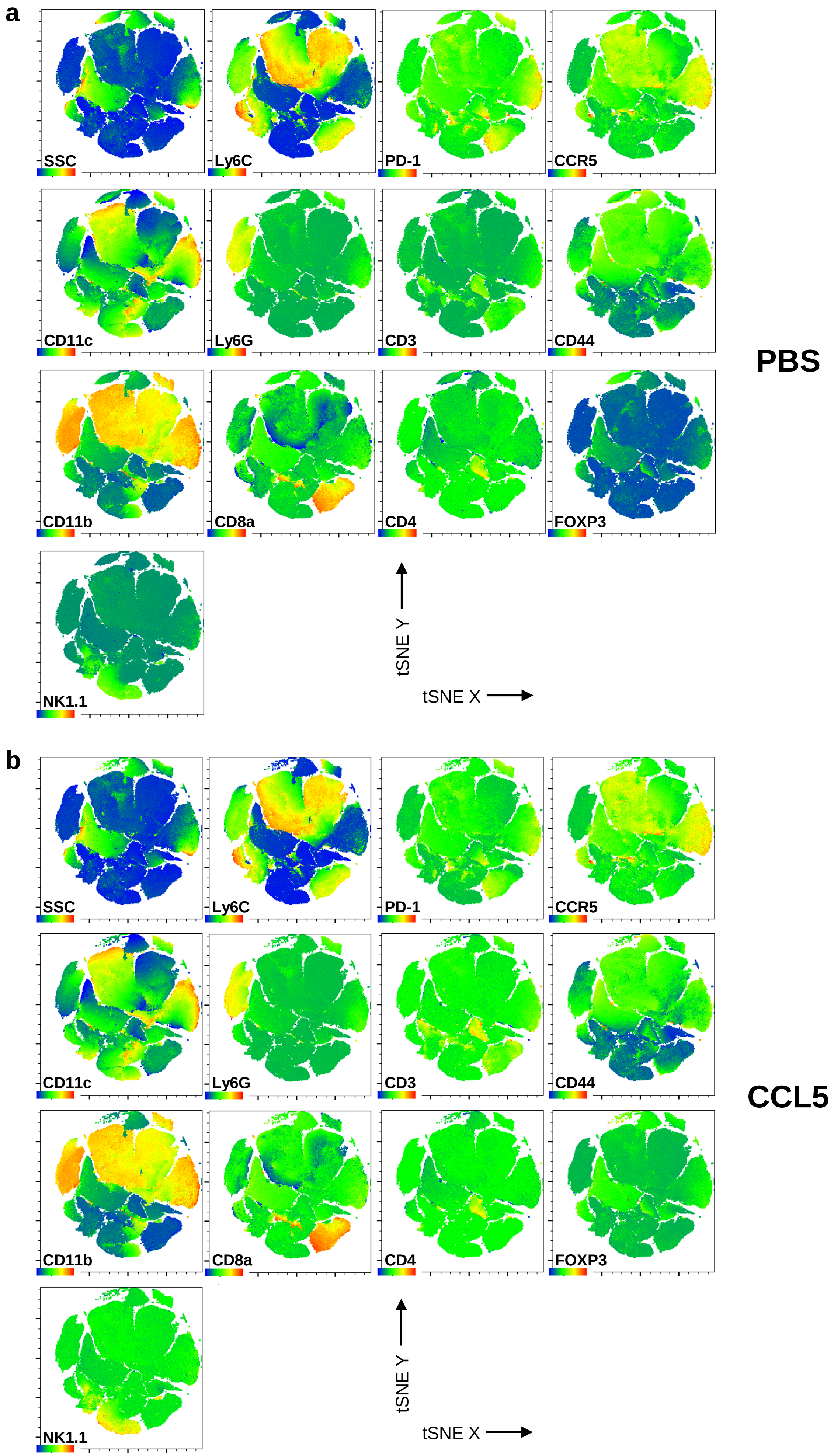

Figure S8. Flow cytometry-based expression of key genes in tSNE identified populations. Plots show heat maps of the indicated marker for each parameter used in tSNE analysis in PBS control **(a)** or recombinant CCL5 **(b)** treated samples.
